# Does China’s Urban Development Satisfy Zipf’s Law? A Multiscale Perspective from the NPP-VIIRS Nighttime Light Data

**DOI:** 10.3390/ijerph17041460

**Published:** 2020-02-24

**Authors:** Yizhen Wu, Mingyue Jiang, Zhijian Chang, Yuanqing Li, Kaifang Shi

**Affiliations:** 1 School of Geographical Sciences, State Cultivation Base of Eco-agriculture for Southwest Mountainous Land, Southwest University, Chongqing 400715, China; wyz19981013@email.swu.edu.cn (Y.W.); acebaby@email.swu.edu.cn (M.J.); pdszjchang@gmail.com (Z.C.); xslyq@foxmail.com (Y.L.); 2 Chongqing Jinfo Mountain Field Scientific Observation and Research Station for Kaster Ecosystem, School of Geographical Sciences, Southwest University, Chongqing 400715, China; 3 Chongqing Engineering Research Centre for Remote Sensing Big Data Application, School of Geographical Sciences, Southwest University, Chongqing 400715, China

**Keywords:** nighttime lights, urban development, Zipf’s law, multiscale analysis, China

## Abstract

Currently, whether the urban development in China satisfies Zipf’s law across different scales is still unclear. Thus, this study attempted to explore whether China’s urban development satisfies Zipf’s law across different scales from the National Polar-Orbiting Partnership’s Visible Infrared Imaging Radiometer Suite (NPP-VIIRS) nighttime light data. First, the NPP-VIIRS data were corrected. Then, based on the Zipf law model, the corrected NPP-VIIRS data were used to evaluate China’s urban development at multiple scales. The results showed that the corrected NPP-VIIRS data could effectively reflect the state of urban development in China. Additionally, the Zipf index (*q*) values, which could express the degree of urban development, decreased from 2012 to 2018 overall in all provinces, prefectures, and counties. Since the value of *q* was relatively close to 1 with an *R*^2^ value > 0.70, the development of the provinces and prefectures was close to the ideal Zipf’s law state. In all counties, *q* > 1 with an *R*^2^ value > 0.70, which showed that the primate county had a relatively stronger monopoly capacity. When the value of *q* < 1 with a continuous declination in the top 2000 counties, the top 250 prefectures, and the top 20 provinces in equilibrium, there was little difference in the scale of development at the multiscale level with an *R*^2^ > 0.90. The results enriched our understanding of urban development in terms of Zipf’s law and had valuable implications for relevant decision-makers and stakeholders.

## 1. Introduction

Global cities have entered a stage of rapid development. The growth of an urban area is reflected in its significant changes, such as gross domestic product (GDP) growth, population increases, urban built-up area expansion, and environmental pollution [1,2]. As one of the largest developing countries in the world, China has experienced the largest and fastest urban development in the history of the world [3]. For instance, by 2017, the urban and rural settlement areas as a whole covered 209,950 km^2^, nearly 13.6 times the 15,364 km^2^ mapped in 1978 [4]. The magnificent speed of urban development of the kind seen in China is rare and world-leading [5,6,7]. Therefore, an in-depth analysis of China’s urban development is conducive to the implementation of a global sustainable development strategy. According to the “National New Urbanization Planning (2014–2020)” released by the state council in 2014, the urbanization rate of permanent residents should reach about 60% by 2020, which is important information from the perspective of urban strategy in China [8]. If the urbanization rate exceeds 60%, the population will accelerate the migration to metropolitan areas, especially to central cities directly under the control of the central government, provincial capitals, and cities under separate state planning [9]. At present, China’s urbanization has entered a stage in which central cities drive urban agglomeration and thus regional economic development. This stage will be maintained for quite a long time in the future. Reasonable adjustment of the urban development process and urban development layout will be conducive to China’s future sustainable development. However, there are different opinions about the arrangement of the urban development layout during this period of rapid urban development in China. Hence, an in-depth analysis of China’s urban development is conducive to understanding the pattern of urban development and formulating accurate policy guidance in China to guide rational urban development for sustainable development.

**Figure 1:**
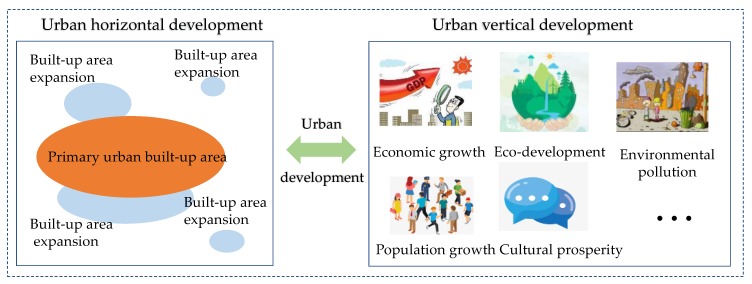
Framework of urban development.

Urban development involves the changing and growing process of the status, function, attraction, and radiation forces of an urban area, which are mutually reinforcing. It is a complex changing process of complex systems, including the population, society, economy, ecology, land, culture, and other subsystems [10,11,12]. Horizontal expansion and vertical improvements are deployed in urban development (Figure 1). A horizontal expansion is represented by an increase in the number and scale of cities and the expansion of the urban built-up area, i.e., improvements in the urbanization level and urban size [13,14,15,16]. A vertical improvement represents an increase in the population and GDP growth, i.e., the socio-economic and environmental development of cities [17,18,19,20].

Urban development has been found to follow Zipf’s law. It says that if all cities in a certain region are ranked according to the size of their population, the sizes of the cities are inversely proportional to their rank, that is, the product of the rank of any city and its population size is equal to the size of the population of the first city in a certain region. Auerbach and Zipf first proposed that the empirical relationship between the city’s ranking and population size in a real urban system conforms to Zipf’s law [21]. Some existing studies have investigated whether urban development satisfies Zipf’s law from different perspectives, such as GDP, built-up areas, and population [22,23,24]. For example, Deng et al. [25] explored China’s urban built-up area with Zipf’s law to explain that the built-up areas of all cities in China had maintained a growth trend, but the rate of expansion varies greatly from period to period. Census data from the National Institute of Statistics were combined with the population and GDP to estimate the Zipf’s index [26,27,28,29]. Previously, Wen et al. [30] showed that China’s urban scale distribution was relatively balanced, virtually conforming to Zipf’s law by using China’s urban population data from 1990 to 2010 and a double-logarithmic regression model to test China’s urban scale and urban rank through Zipf’s law. However, many problems were still unsolved using traditional data because of the long lag time from generation to analysis, the large cost, the lack of digital form and spatial information, different levels of details on different scales, and the incapability of showing the full picture. There was a long lag time between when the written statistical data were generated and when they were available to the public. For instance, the authoritative data for China’s population statistics were the census, but the data were collected only once every 10 years, which made it difficult to track the population changes of large, medium, and small cities over the years. Additionally, the built-up area data were normally obtained from traditional remote sensing data. High- and medium-spatial-resolution remotely sensed images, such as those captured by the Landsat Thematic Mapper (TM) and the IKONOS satellite, the Sentinel-2 satellite, has been exploited in a variety of studies to characterize urban built-up areas to study urban development and processes [31,32]. However, such studies were cost-intensive given their limited geographic coverage and required a large amount of time and human resources to extract the urban information for a large region. Furthermore, the GDP and population data were written statistical data, which had deficiencies regarding digital analysis. The data also lacked adequate spatial details because statistical yearbook data, such as for GDP and population, used for urban development research were usually collected at the administrative unit scale. Moreover, most statistical yearbook data were openly published at the provincial and municipal levels, while county data were not, leaving the different levels of detail at different spatial scales. Lastly, although the GDP, population, and built-up area could describe the development of the city, they were only a part of the information on urban development, reflecting only one aspect each. Thus, they cannot show the full picture of the urban development of cities in China.

The nighttime light time-series images provide a new perspective on the spatiotemporal changes for urban development [33,34]. The nighttime light data can detect urban nighttime lights and even low-intensity lights emitted by small-scale residential areas and traffic flows. The nighttime light data can reflect the degree of urban development, such as the population concentration, urbanization, economic activity, and other socio-economic aspects, and then reflect the socio-economic conditions of a country and the actual urban development situation [35,36,37]. Since nighttime light data can reflect the socio-economic and other relative activities of human beings well, and the activities related to various aspects of urban development are closely related to social and economic activities, nighttime light data can be used for more accurate for urban development monitoring, which has been proved by scholars at home and abroad. For example, Amaral et al. [38] analyzed nighttime light imagery as an information source to detect human settlements and to estimate the urban population in the Amazon region, as well as identify the potential ability of nighttime light images for estimating urban population and the technical limitations of using such images as a way to annually monitor urban population dynamics in a region. Shi et al. [39] combined nighttime light images and statistical data to assess spatiotemporal variations in urban CO_2_ emissions in China. Yu et al. [40] proposed a preprocessing method that applied a logarithmic transformation to the original nighttime light composite data for urban built-up area extraction. Shi et al. [41] applied linear regression to fit the correlation between the total nighttime light (TNL) data and the EPC (electricity power consumption) and GDP at provincial- and prefectural-level divisions. Additionally, many other studies discussed PM_2.5_ emissions [42,43], population [35,44], EPC [45], and other fields related to urban development. Hence, the nighttime light data provides a comprehensive way to study urban development in the form of a spatiotemporal pattern. Compared with the first-generation nighttime light data, i.e., the Defense Meteorological Satellite Program’s Operational Linescan System (DMSP-OLS) data, the national polar-orbiting partnership’s visible infrared imaging radiometer suite (NPP-VIIRS) nighttime light data’s spatial resolution, temporal resolution, and radiation resolution has been greatly improved, and these data were more suitable for the study of human economic and social activities.

Current studies focusing on Zipf’s law in urban development were from only a single or large scale. For instance, Small et al. [46] compared size–frequency distributions with size–area distributions and illustrated the effect of detection frequency thresholds on the number of contiguous lights and total lighted area to explore urban extent. Li et al. [47] analyzed the spatial and temporal patterns of national urban development for countries along the One Belt And One Road line, and Jiang et al. [20] analyzed whether Zipf’s law describes global natural cities. Huang et al. [48] analyzed city-size evolution at the city level based on nighttime light data at the national level and demonstrated that the nighttime light data and the Zipf’s law method were effective at uncovering urban development dynamics more consistently from both national and city perspectives. Many studies have also explored whether Zipf’s law describes global natural cities using nighttime light data [49]. Other studies have used nighttime light data to investigate Zipf’s law for urban agglomerations or prefectures [25,50]. However, there is a lack of multiscale spatial-temporal research on urban development in China. Zipf’s law on a single scale is not always consistent with that on other scales; therefore, the results of urban development on other scales are unknown. Although there have been extensive studies on urban development from different perspectives, little is known about China’s urban development at different levels. However, the problem is that most previous studies were conducted on a single spatial scale or in a single city with various geographical and political contexts. Analysis of data at different scales yields different results. Different scales have different influence mechanisms. In the field of geography, the influence of the selected area unit on the analysis result was defined as the modifiable areal unit problem (MAUP) [51], which is one of the main reasons for uncertainty in spatial data analysis results. Theoretically, the current basic administrative divisions of the People’s Republic of China are divided into three levels in mainland China: province, prefecture, and county. China has multiple dimensions of geographically administrative divisions. The temporal and spatial variations of urban development reflect the evolution of urban development in time and space. As the administrative area cannot completely describe the city size and the implementation of the urban development strategy policy main body, to achieve the evaluation of urban development, it is necessary to analyze the different administrative scales of urban development in line with the differentiation of urban development characteristics in different regions for the development of policies and measures. Currently, most studies have preferred to focus on the dynamic spatiotemporal variations of an administrative region, and thus multiscale research on urban development is lacking. In particular, the study of the dynamics of the national urban development spatiotemporal pattern has been mostly at the provincial level, while the smaller-scale studies, such as those focusing on the prefecture-level and county-level urban development spatiotemporal patterns, have been ignored. Spatial data had multi-granular and multiscale characteristics, and the relationship between the attribute data often changes with the research granularity and regionalization. When spatial analyses were carried out on the same geographic area with different resolutions or different scales, inconsistencies often occurred, which is called the scale effect [48]. The scale effect has become a central topic in geographical research. The space scale and the developmental trends of the geographical phenomena behind the influencing factors and mechanisms are likely to be different due to different spatial scales. Especially for the administrative regional scale, higher administrative units, more often than low-level administrative units, have more administrative, economic, and financial rights, leading to the urban development at different administrative scales presenting different time and space distribution patterns. The multiscale viewpoint has been demonstrated to be effective in many empirical analyses, such as regional economics, ecosystem management, and metropolitan governance. Specifically, it was worth noting that higher administrative units generally had stronger powers (e.g., financial, fiscal, and cultural powers) than lower administrative units in China, which likely led to differential impacts on both the scope and scale of urban development.

In summary, the following specific research questions remained unsolved: (1) Does Zipf’s law hold in China’s urban development? (2) What were the differences in Zipf’s law for China’s urban development from a multiscale perspective? To address the above questions, we selected 31 Chinese provincial cities, 333 Chinese prefectural cities, and 2845 Chinese county-level cities as study cases and conducted experiments using the NPP-VIIRS data. First, the NPP-VIIRS data was corrected to remove outliers to accurately reflect China’s urban development. Next, in combination with the Zipf’s law model, this study compared the urban development at three different scales, i.e., the province-level, prefecture-level, and county-level scales. The paper offers a scientific and effective way to deepen our understanding of the urban development according to Zipf’s law in Chinese cities at multiple scales and suggests a rational path for the government to improve the quality of urban development through sustainable urban planning.

## 2. Study Area and Data Sources

### 2.1. Study Area

**Figure 2:**
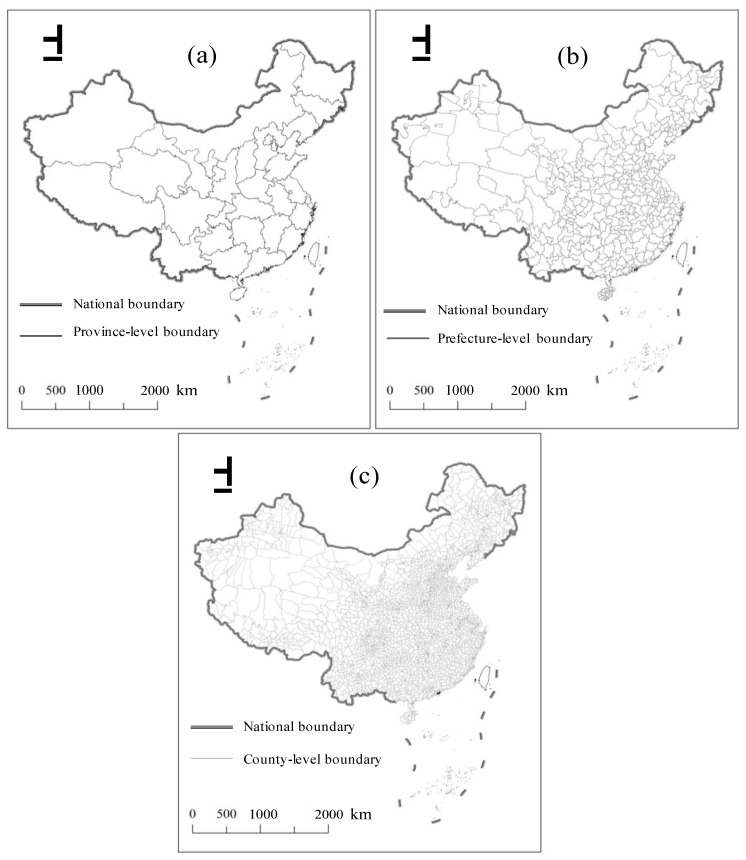
Maps of the study areas: (**a**) provinces, (**b**) prefectures, and (**c**) counties.

This study selected China’s mainland as the study area, excluding the Hong Kong, Macao, and Taiwan provinces because of insufficient relevant statistical data. China’s administrative divisions have a spatial hierarchical structure. The socio-economic policies are conveyed through multiple levels of government, including the provincial, prefecture, and county levels [42,52]. Currently, there are 31 province-level regions, including 22 provinces, 5 autonomous regions, and 4 municipalities (Beijing, Shanghai, Tianjin, and Chongqing) (Figure 2). Thus, province-level units, prefecture-level units, and county-level units were taken as the basic units to study the regional differences and spatial patterns for performing a multiscale analysis of China’s urban areas. It was noted that the areas of the four municipalities were smaller than the other provinces and autonomous regions. Consequently, Chongqing was merged into Sichuan to become part of the Sichuan prefecture, Beijing and Tianjin were merged into Hebei, and Shanghai was merged into Jiangsu to maintain the integrity of the embedded structure. Therefore, 27 province-level units, 333 prefecture-level units, and 2845 county-level units were taken as the objects of this study at the national level.

### 2.2. Data Sources

The NPP-VIIRS data, the DMSP-OLS data, the administrative vector boundary, and statistical data (population, GDP, EPC) were used in this study.

**Figure 3:**
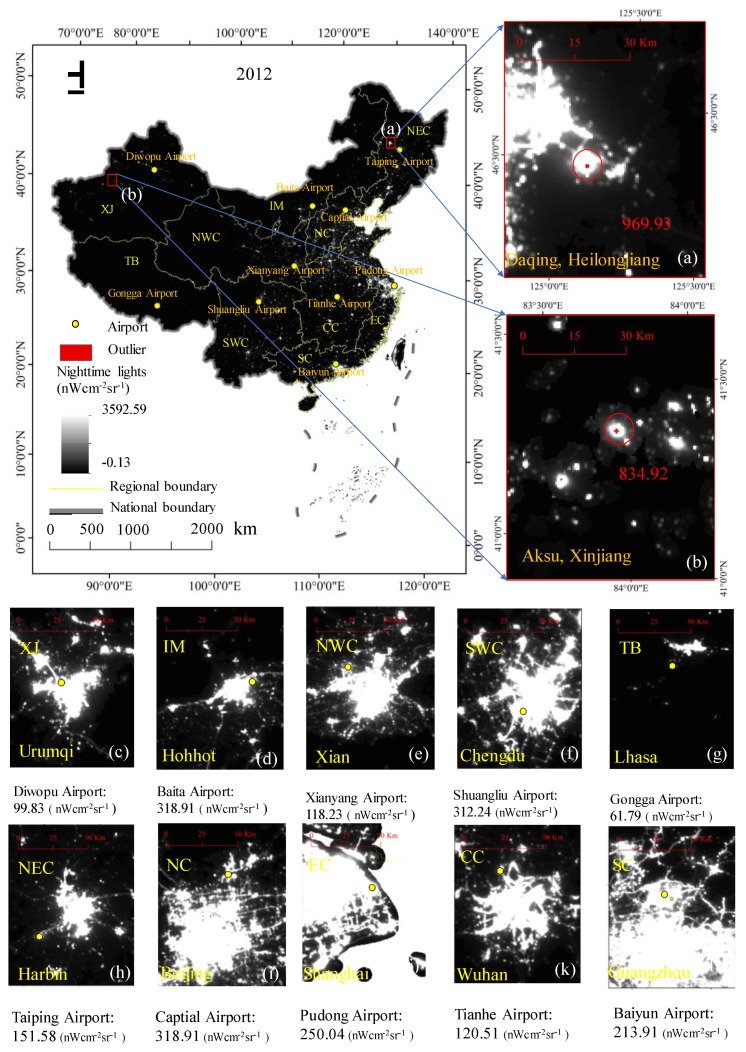
The original National Polar-Orbiting Partnership’s Visible Infrared Imaging Radiometer Suite (NPP-VIIRS) data in 2012. (**a**) outliers in Daqing, Heilongjiang; (**b**) outliers in Aksu, Xinjiang. (**c**) the airport in Urumqi; (**d**) the airport in Hohhot; (**e**) the airport in Xian; (**f**) the airport in Chengdu; (**g**) the airport in Lhasa; (**h**) the airport in Harbin; (**i**) the airport in Beijing; (**j**) the airport in Shanghai; (**k**) the airport in Wuhan; (**l**) the airport in Guangzhou.

The NPP-VIIRS nighttime light monthly composite data with an image resolution of 15 arc-second geographic grids (approximately 500 m) from 2012 to 2018 were obtained from the National Oceanic and Atmospheric Administration’s National Geophysical Data Center (NOAA/NGDC) website, which are available free of charge for users. Figure 3 shows the original NPP-VIIRS data. The Version 4 DMSP-OLS nighttime stable light yearly composite data with an image resolution of approximately 30 arc seconds (approximately 1 km) for 2012 were used in this study. This dataset was also downloaded from the NOAA/NGDC website (https://ngdc.noaa.gov/eog/index.html). The vector boundary data of the administrative divisions of mainland China, including the national, provincial, prefectural, and county boundaries, were obtained from the National Geomatics Center of China. The socio-economic indicators, which included the total population in each province, the GDP, and EPC, were obtained from the China Statistics Yearbook, 2012–2017. Unfortunately, the China Statistics Yearbook, 2018 was not yet available.

To maintain spatial consistency, all the spatial data were projected into an Albers equal-area conic projection with reference to the WGS84 datum and resampled to a spatial resolution of 500 m based on a nearest-neighbor resampling algorithm (excluding administrative boundaries). The nighttime light images of mainland China were extracted from the DMSP-OLS and NPP-VIIRS global datasets by using a polygon-shaped mask of the national boundary of China with a 5 km buffer.

## 3. Methods

### 3.1. Correction of the NPP-VIIRS Data

The NPP-VIIRS data were from the second-generation nighttime light data products, which were released in 2012. Compared with the first-generation nighttime light data, DMSP-OLS data, the NPP-VIIRS data had some advantages. First, as a new generation of nighttime light data, the spatial resolution, temporal resolution, and radiation resolution of the NPP-VIIRS data had been greatly improved; therefore, these data were more suitable for the study of human economic and social activities. Additionally, global nighttime lights were detected using the same sensor, making nighttime lights comparable across different regions. Since April 2012, the NPP-VIIRS data has been updated quickly and is released once a month, which is conducive to real-time monitoring of global urban development and dynamic assessment of the urban scale distribution. Second, the NPP-VIIRS data employs onboard calibration (not available for the DMSP-OLS data), which increases the data quality. Third, the wide-angle radiation detector of NPP-VIIRS eliminates the phenomenon of light oversaturation and enhances the detection sensitivity; additionally, there is no light saturation, since a wider radiometric detection range is used. The VIIRS day/night band on the Suomi NPP has a specified dynamic range of approximately seven orders of magnitude, i.e., from 3 × 10^−9^ W·cm^−2^·sr^−1^ to 0.02 W·cm^−2^·sr^−1^, such that the sensors can detect even fainter lights. Different from DMSP-OLS, which discarded the effects of sunlight, glare, moonlight, clouds, and auroras, the NPP-VIIRS datasets obtained from NOAA is not filtered to screen out lights from auroras, snow reflection, fires, boats, other temporal lights, and background noise. The noise in the datasets affects the accuracy of the data regression between GDP and total nighttime light data of NPP-VIIRS or the population and total nighttime light data of NPP-VIIRS; therefore, it cannot reflect the true situation regarding human activity and socio-economic activity. For example, some of the nighttime light values were extremely abnormal in the original 2012 NPP-VIIRS data (Figure 3).

The steps for removing the background noises and abrupt bright spots in the original NPP-VIIRS data were as follows. First, the NPP-VIIRS monthly composite data in some areas of China were missing to varying degrees; however, the monthly data for the whole year, except April to August, were available for the study from 2012 to 2018. Thus, the residual monthly NPP-VIIRS data for China in the same year were used to calculate the average radiance value for each grid pixel to obtain the yearly composite mean NPP-VIIRS data for each pixel. Meanwhile, the residual monthly image data for China in the same year were used to find the maximum value of the pixel in the same position to obtain the yearly composite maximum NPP-VIIRS data. In the next steps, the two types of original yearly composite data were called the NPP-VIIRS data. Second, the DMSP-OLS data had a spillover effect such that they showed a larger lit area than the NPP-VIIRS data, which could remove the nonhuman activity lights to some extent. Therefore, it could be assumed that most of the bright areas of the NPP-VIIRS data would be retained in the DMSP-OLS data [38]. Next, a mask containing all pixels with positive digital number (DN) values from the DMSP-OLS data in 2012 was extracted. Then, the mask multiplied the original NPP-VIIRS data to generate the original NPP-VIIRS data. Because the lit areas detected by the DMSP-OLS data were consistently larger than those of the NPP-VIIRS data, it was believed that all of the lit areas related to human activity were maintained in the primary corrected NPP-VIIRS data [53]. Due to the lack of explanation in the metadata, such negative values were regarded as background noises caused by the data composition processes. In addition, the pixels with negative values in the NPP-VIIRS data were assigned values of 0. Then, the initial corrected data could already provide slightly better information for urban development than the uncorrected data, but we still detected a few outliers in the northeastern and western areas of China, such as Daqing in Heilongjiang (Figure 3a) and Aksu in Xinjiang (Figure 3b), via a visual inspection. The two sampling areas bounded by red rectangles in Figure 3 were magnified and the outliers higher than the surrounding value were circled and marked in red. The abnormal values of the sampling area were well above the threshold for this region. The outliers were probably caused by the lights from the fires of oil or gas wells located in those areas. Therefore, due to the regional differences in socio-economic status, atmospheric conditions, and landscape, a unique threshold could not completely remove the remaining outliers for all the regions in China. As such, we subdivided China into 10 regions, including Northeast China (NEC), Inner Mongolia (IM), Southwest China (SWC), Tibet (TB), South China (SC), Xinjiang (XJ), Northwest China (NWC), North China (NC), East China (EC), and Central China (CC) (Figure 2). As is known, the brightest place in a city is its airport, so the airport lights’ intensity was the brightest part of a city. Therefore, we selected the most developed metropolitan airport in each region and identified its maximum light intensity value as the maximum value for all pixels in each region (Figure 2). The optimal threshold value for each region was determined based on the maximal value of the largest representative cities in the specific region. We also selected the light intensity value of the largest airport in each region to correct the outliers in each region (Figure 3). The final corrected NPP-VIIRS data were generated from the maximum value of each region. Each pixel whose value was larger than the maximum value of its region in the NPP-VIIRS data was assigned a new value, i.e., the maximum value from its neighboring pixels. The new value was the maximal value within the pixel’s immediate eight neighbors. If the values of the immediate eight neighbors were all larger than the maximal value in this region, the maximal value of the eight neighbors for each pixel in the immediate eight neighbor area was selected. After this process, all the pixel values in the corrected NPP-VIIRS data were smaller than the maximum value of its region.

To verify the accuracy, we compared the original value and the corrected value of the two sampling areas via visual interpretation. To check the robustness of the corrected NPP-VIIRS data results, we used the nighttime light values of each province of the corrected NPP-VIIRS data and the GDP, EPC, and population statistical data in a linear regression from 2012 to 2017.

### 3.2. Zipf ’s Law

The relationship between the size of a city and the rank of the city relative to all the cities in the country is described by Zipf’s law, which was named after the linguist George Kingsley Zipf. In 1949, Zipf applied the power law to various aspects of linguistics and economic geography and found that the natural and human society followed the rank–size law according to in the principle of least effort. Then, Zipf analyzed the relevance of the city’s size and its rank and revealed that they fit the rank–size law. Later, the regularity between a city and its rank became known as the famous Zipf’s law. The Zipf’s law was not universal, but it had been accepted by many as an ideal state of equilibrium. Catterow’s formula was one of the most common expressions of Zipf’s law, shown as follows:

(1) $$S_{i}=S_{1}\cdot R_{i}^{-q}$$

To improve the ease of interpretation, a natural logarithm transformation is usually performed on the Catterow formula, as follows:

(2) $$ln\left(S_{i}\right)=ln\left(S_{1}\right)-qln\left(R_{i}\right)$$

where *q* is the Zipf index (hereinafter referred to as *q*), which reflects the concentration of the urban development distribution; *R* is the rank of a city’s total nighttime light (sorted according to descending TNL); *S*_1_ is the degree of urban development of the largest city in theory; and *S_i_* is the degree of urban development of a city of rank *i*. When *q* ≈ 1, the urban development distribution is said to satisfy Zipf’s law such that the second city in a country had half the size of the largest city, the third city had one-third of the size of the largest city, and so on, which indicated that in the urban system, the cities in the middle, high, and low orders develop harmoniously, and the urban development is in a state that satisfies the Zipf’s law. When the value of *q* is close to 1, it means that urban development is close to the ideal state. When *q* < 1, it suggests that in the urban system, compared to large cities, the nighttime lights are concentrated mainly in medium and small cities, that is, the monopoly status of the urban development of the primate city or large cities was not strong enough and the medium and small cities develop relatively well. This means that a relatively evenly balanced urban development distribution exists. When *q* > 1, it means that in the urban system, urban development is concentrated mainly in large cities and the development of medium and small cities is relatively insufficient. Moreover, the greater *q* is, the stronger the monopoly capacity of the urban development of the primate city was. Notably, if *q* approaches ∞, there is only one city in the urban system; if *q* = 0, the sizes of all the cities are even in the urban system. The closer the *q* is to 1, the more likely it was that Zipf’s law holds [54]. A long time-series dynamic analysis was performed for this region; when the *q* value increased gradually, it indicated that the distribution of urban development and nighttime lights tended to be concentrated rather than dispersed. When the *q* value decreased gradually, it indicated that the power of the urban development distribution and nighttime lights tended to be dispersed rather than concentrated. The smaller the value of *q* was, the smaller the development gap between cities was, and the more balanced the urban development scale system was. The larger the value of *q* was, the greater the development gap between cities was, and the more polarized the urban development scale system was.

In this study, the TNL of a province, prefecture, and county indicated the degree of its urban development. The rank of the city was based on the total light intensity value in the area. The *q* value was predicted using the ordinary least squares method (OLS). The size distribution of urban development in China at multiple scales was assessed in terms of whether they followed Zipf’s law.

## 4. Results and Discussion

### 4.1. Evaluating the Corrected Results of the NPP-VIIRS Data

**Figure 4:**
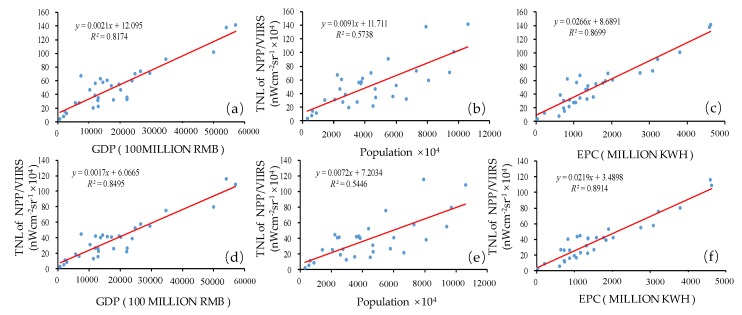
The scatter diagram of the linear regression analysis at the provincial scale. TNL: total nighttime light. Linear regression of (**a**) the corrected NPP-VIIRS maximum data and GDP; (**b**) the corrected NPP-VIIRS maximum data and population; (**c**) the corrected NPP-VIIRS maximum data and EPC; (**d**) the corrected NPP-VIIRS mean data and GDP; (**e**) the corrected NPP-VIIRS mean data and population; (**f**) the corrected NPP-VIIRS mean data and EPC.

To verify the accuracy of two types of corrected NPP-VIIRS data, we used GDP, population, and EPC data to perform a regression with the corrected NPP-VIIRS mean data and the corrected NPP-VIIRS maximum data at the provincial scale. The regression results of the *R*^2^ of the NPP-VIIRS mean data and the NPP-VIIRS maximum data with socioeconomic statistical indicators from 2012 to 2017 are shown in Table 1. Figure 4 also straightforwardly describes the fitting of two types of data and three types of statistical data separately. From Table 1, the average *R*^2^ of the corrected NPP-VIIRS mean data and GDP was 0.84, while that of the corrected NPP-VIIRS maximum data and GDP was 0.78. The average *R*^2^ of the corrected NPP-VIIRS mean data and electricity consumption was 0.89, while that of the corrected NPP-VIIRS max data and electrical power consumption was 0.86. Additionally, the average *R*^2^ of the corrected NPP-VIIRS mean data and population was higher than that of the corrected NPP-VIIRS maximum data and population. In total, the results indicated that the corrected NPP-VIIRS mean data could better reflect the reality of mainland China’s urban development in the multiscale analysis. Hence, this study selected the corrected NPP-VIIRS mean data as the corrected NPP-VIIRS data for the rest of the study.

**Figure 5:**
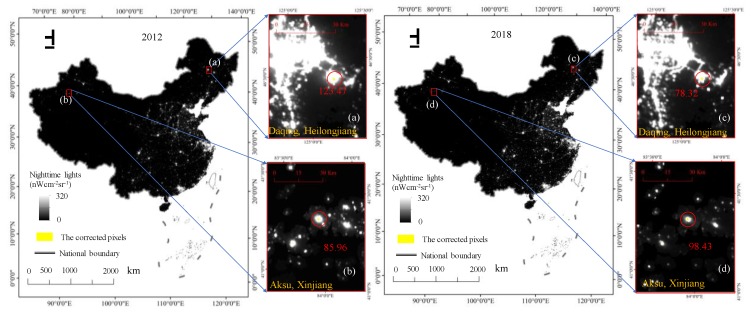
The corrected NPP-VIIRS data in China. (**a**) the corrected valve in Daqing, Heilongjiang in 2012; (**b**) the corrected valve in Aksu, Xinjiang in 2012; (**c**) the corrected valve in Daqing, Heilongjiang in 2018; (**d**) the corrected valve in Aksu, Xinjiang in 2018.

**Figure 6:**
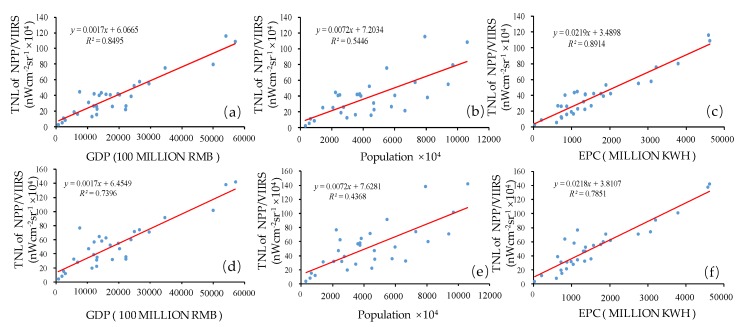
The scatter diagram of the linear regression analysis at provincial scale. Linear regression of (**a**) the corrected NPP-VIIRS data and GDP; (**b**) the corrected NPP-VIIRS data and population; (**c**) the corrected NPP-VIIRS data and EPC; (**d**) the original NPP-VIIRS data and GDP; (**e**) the original NPP-VIIRS data and population; (**f**) the original NPP-VIIRS data and EPC.

Figure 5 shows the corrected NPP-VIIRS data for China in 2012 and 2018. The outliers whose radiance values were larger than the airports of the representative cities in each region in the initial NPP-VIIRS data of mainland China were all removed and modified to the new values determined by its surrounding pixel values. As a verification, we compared the corrected values of two sampling areas and marked the uncorrected outliers in yellow (Figure 5). In Figure 3, the value of the outliers in Daqing, Heilongjiang, was 963.93 nWcm^−2^sr^−1^ and in Aksu, Xinjiang, it was 834.92 nWcm^−2^sr^−1^. However, the maximal value of Northeast China was 151.58 nWcm^−2^sr^−1^, and the maximal value of Xinjiang was 99.83 nWcm^−2^sr^−1^. The value of Daqing, Heilongjiang, after the correction was 123.47 nWcm^−2^sr^−1^ in 2012 (Figure 5a) and 78.32 nWcm^−2^sr^−1^ in 2018 (Figure 5c). The value of Aksu, Xinjiang, after the correction was 85.96 nWcm^−2^sr^−1^ in 2012 (Figure 5b) and 98.43 nWcm^−2^sr^−1^ in 2018 (Figure 5d). Compared to the original NPP-VIIRS data in 2012, the value of Daqing, Heilongjiang, was 963.93 nWcm^−2^sr^−1^ and that of Aksu, Xinjiang, was 834.92 nWcm^−2^sr^−1^. Therefore, after the correction, the outliers were eliminated. Then, the corrected data compared with the original data through the linear regressions, the TNL–GDP, TNL–population, and TNL–EPC relationships at the provincial level were evaluated with the corrected 2012 composite nighttime light data, which were generated by the immediate eight neighbors and the original 2012 composite nighttime light data (Figure 6). The regression results of the *R*^2^ of the corrected NPP-VIIRS data and the original NPP-VIIRS data with socioeconomic statistical indicators from 2012 to 2017 are shown in Table 1. The *R*^2^ of the corrected NPP-VIIRS data and GDP was 0.85 (Figure 4a), whereas that of the TNL from the original NPP-VIIRS data and GDP was 0.74 (Figure 4d). The *R*^2^ of the corrected NPP-VIIRS data and population was 0.55 (Figure 4b); however, that of the TNL from the original NPP-VIIRS data and population was 0.44 (Figure 4e). The *R*^2^ of the corrected NPP-VIIRS data and EPC was 0.89 (Figure 4c), which was higher than the *R*^2^ of the TNL from the initial corrected NPP-VIIRS data and EPC (Figure 4f). Overall, the fit of the corrected NPP-VIIRS data to the socio-economic statistics was higher than that of the original data. Compared with the original data, the corrected NPP-VIIRS data better reflected the reality of the socio-economic information for China.

### 4.2. Analyzing the Zipf ’s Law Results of China’s Urban Development at Multiple Scales

#### 4.2.1. Urban Development at the Provincial Level

**Figure 7:**
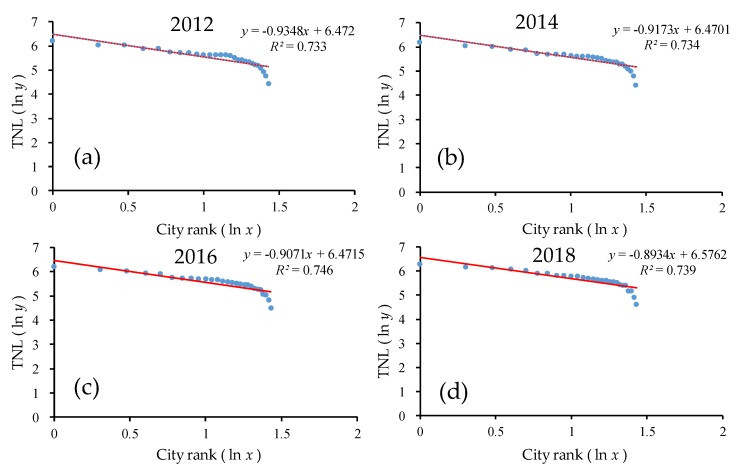
The ln–ln plot of the rank–size of the urban development for all provinces. Line regression of (**a**) TNL and ranking of all provinces in 2012; (**b**) TNL and ranking of all provinces in 2014; (**c**) TNL and ranking of all provinces in 2016; (**d**) TNL and ranking of all provinces in 2018.

As shown in Figure 7 and Table 2, the goodness of fit *R*^2^ of the double-logarithm regression model for the provincial scale was greater than 0.70 for all provinces from 2012 to 2018, thus passing the significance test. In the double-logarithm regression model, the value of *q* was less than but close to 1.0. The provincial-level *q* value decreased significantly from 0.93 in 2012 to 0.89 in 2018, showing a decreasing trend with a small fluctuation overall. On the one hand, the result indicates that urban development was close to the ideal state (*q* ≈ 1), and urban development mainly depended on the middle-, high-, and low-order provinces and developed harmoniously at the provincial level. This result suggests that in the urban system, the power of development was found throughout the large, medium, and small provinces, i.e., the monopoly status of the large, medium, and small provinces developed relatively well. On the other hand, the continuous decrease of the *q* value indicates that the overall trend of the urban development scale on the provincial scale in China was increasingly dispersed, and the power of the urban development scale distribution tended to be dispersed more than it was concentrated. The urban system changed from decentralized to more decentralized. The provinces in the lower rank developed rapidly, while those in the higher rank developed slowly.

As seen from Figure 7 in the ln-ln model, there was an obvious trailing phenomenon, which deviated significantly from the regression line. Furthermore, the rear part of the scale distribution curve of the urban development level obviously deviated from Zipf’s law. The main reason was that the economic development of Tibet, Qinghai, and the other provinces in the country was relatively backward, and the level and scale of urban development was weak. Furthermore, the majority of the western population was pouring into the eastern region, resulting in those provinces having weaker development [55].

**Figure 8:**
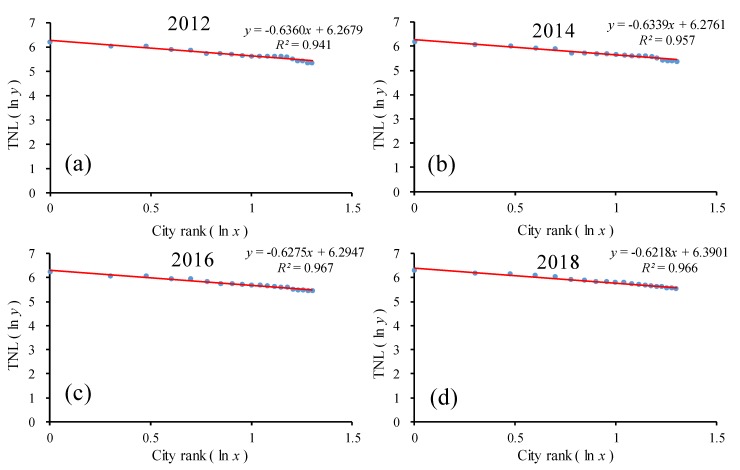
The ln-ln plot of the rank–size of the urban development for the top 20 provinces. Line regression of (**a**) TNL and ranking of the top 20 provinces in 2012; (**b**) TNL and ranking of the top 20 provinces in 2014; (**c**) TNL and ranking of the top 20 provinces in 2016; (**d**) TNL and ranking of the 20 provinces in 2018.

Therefore, on the same scale, this study chose the top 20 provinces in China for the time-series analysis. As shown in Figure 8 and Table 2, the goodness of fit *R*^2^ of the double-logarithm regression model for the provincial scale was greater than 0.90 in the top 20 provinces from 2012 to 2018, which passed the significance test. The goodness of fit was significantly better than all provinces. In the log-log model, the regression line hardly changed from 2012 to 2018. In the double-logarithm regression model, the value of *q* was approximately 0.60. As the values of *q* were all less than 1, this indicates that among the top 20 provinces, the monopolistic nature of the urban development in the first province was not prominent enough, and the level of urban development in the small- and medium-sized provinces was not much different and the number was large, and the urban development scale of each province was evenly distributed, which means that they were in relative equilibrium. Additionally, the overall *q* value showed a decreasing trend with a small fluctuation, i.e., from 0.63 in 2012 to 0.59 in 2017, indicating that the urban development disparity among the top 20 provinces in China was gradually decreasing and the urban development scale was more dispersed than concentrated. The urban system changed from decentralized to more decentralized.

From the above statistical analysis, the development of the top 20 provinces in China was undoubtedly a relative equilibrium distribution type, but all provinces had opposite performances such that they were relatively close to meeting Zipf’s law. Locational advantages had significant effects on urban development after China’s economic reform. The coastal provinces had higher rates of increase in terms of the development level. By contrast, the border cities had lower rates of increase [56]. In all provinces across the country, according to the statistics, Zhejiang’s GDP in 2017 was 1.49 times the GDP of Zhejiang in 2012, Tibet’s GDP in 2017 was 1.87 times the GDP of Tibet in 2012, and Zhejiang’s GDP was 49.45 times that of Tibet in 2012. However, with the development of cities, Zhejiang’s GDP was 39.49 times that of Tibet in 2017. The gap between the two provinces greatly and continuously declined. Meanwhile, in the top provinces, Guangdong’s GDP was 1.65 times that of Zhejiang in 2012, and Guangdong’s GDP was 1.73 times that of Zhejiang in 2017. The gap between the two provinces had increased slightly. The Zhejiang, Jiangsu, and Guangdong provinces had developed well at various scales and levels. There were many large- and medium-sized core cities that drove the local economy, and the small towns were also well developed. The Tibetan autonomous region was sparsely populated, economically backward, and had an underdeveloped urban development system. Therefore, these provinces differed greatly. Whether in all provinces or in the top provinces, the decreasing of the *q* value means that the provinces were growing, with the built-up areas expanding, the population increasing, and the economy growing, which resulted in the gap between the provinces narrowing. The actual scale of China’s top-ranking provinces, especially the largest provinces, was smaller than the theoretical value. Compared to the top 20 provinces, the urban development of all provinces was close to the ideal state [57]. In other words, the distribution of urban development for all provinces was partly considered to be in a natural state of optimal distribution. From the perspective of national policies and the development of China’s provinces, there is still significant room for development. It can be seen that China’s current provincial development strategy focuses on small- and medium-sized provinces, such as Tibet, Qinghai, etc. Therefore, it can be inferred that the Chinese government was vigorously supporting the less-developed provinces and was improving their urban development level, and at the same time, it was also taking into account the development of the former provinces, thus driving the development of the latter provinces through the development of the former provinces.

#### 4.2.2. Urban Development at the Prefectural Level

**Figure 9:**
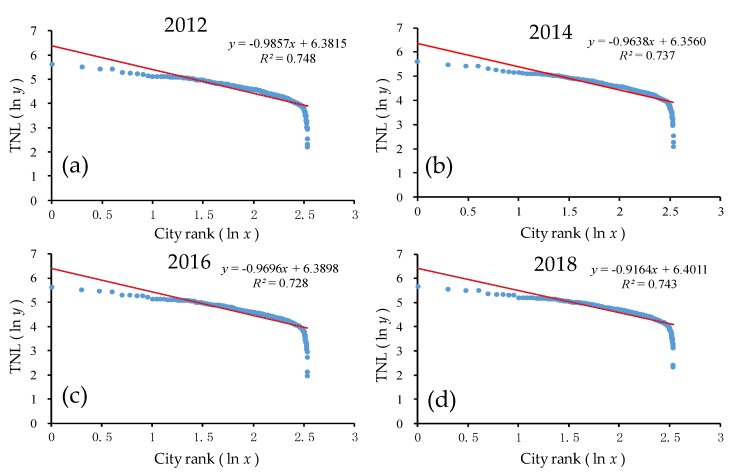
The ln-ln plot of the rank–size of the urban development for all prefectures. Line regression of (**a**) TNL and ranking of all prefectures in 2012; (**b**) TNL and ranking of all prefectures in 2014; (**c**) TNL and ranking of all prefectures in 2016; (**d**) TNL and ranking of all prefectures in 2018.

**Figure 10:**
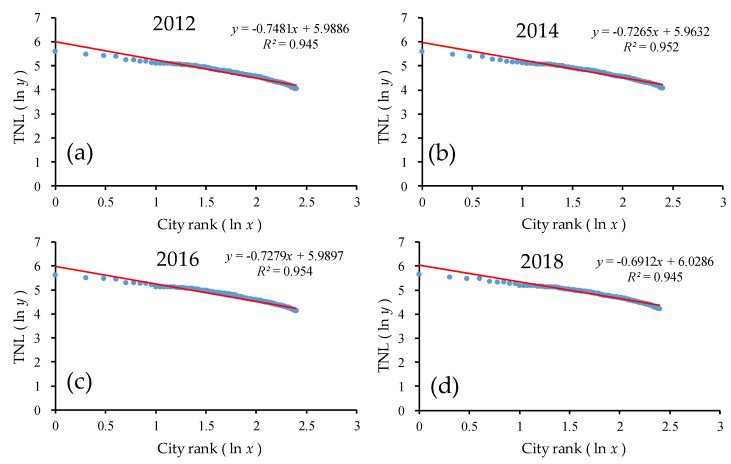
The ln-ln plot of the rank–size of the urban development for the top 250 prefectures. Line regression of (**a**) TNL and ranking of the top 250 prefectures in 2012; (**b**) TNL and ranking of the top 250 prefectures in 2014; (**c**) TNL and ranking of the top 250 prefectures in 2016; (**d**) TNL and ranking of the top 250 prefectures in 2018.

As shown in Figure 9 and Table 3, the goodness of fit *R*^2^ of the double-logarithm regression model for the prefectural scale was approximately 0.70 for all prefectures from 2012 to 2018, which passed the significance test. In the double-logarithm regression model, the value of *q* was near 1.0 but still smaller than 1.0. The change of the *q* value in the prefectural cities fluctuated slightly, from 0.99 in 2012 to 0.86 in 2015, and increased from 0.86 in 2015 to 0.91 in 2018, showing a decreasing trend overall and M-shaped volatility evolution characteristics (Figure 10). On the one hand, this result indicates that urban development was close to the ideal state (*q* ≈ 1), and urban development mainly depended on the middle-, high-, and low-order prefectures that developed harmoniously at the prefectural level, and urban development mainly depended on the medium–low-order cities, while high-order cities did not develop significantly. This finding suggests that in the urban system, the power of urban development was distributed between large, medium, and small prefectures, i.e., the monopoly status of the large prefectures relative to the medium and small prefectures developed relatively well. On the other hand, the decrease of the *q* value from 2012 to 2015 indicated that the overall trend of the urban development on the prefectural scale in China was increasingly dispersed, and the power of the urban development scale distribution tended to be dispersed more than it was concentrated. The urban system changed from decentralized to more decentralized. The cities in the lower rank developed rapidly, while those in the higher rank developed slowly. The increase of the *q* value from 2015 to 2018 indicated that the overall trend of the urban development scale on the prefectural scale in China was increasingly balanced, and the power of the urban development scale distribution tended to be concentrated more that it was dispersed. As the urban system changed from decentralized to centralized, the development speed of the prefectures in the back of the sequence slowed down, while the development speed of the cities in the front accelerated. In general, from 2012 to 2018, the scale of urban development was more dispersed than concentrated.

As seen from Figure 9, in the ln-ln model, there was an obvious trailing phenomenon, which significantly deviated from the regression line, and the rear part of the scale distribution curve of the prefectural development level clearly departed from Zipf’s law. The prefectures located in some western provinces were economically backward and lacked the power to develop, while the prefectures clustered in urban agglomerations had good development momentum.

On the same scale, this study chose the top 250 prefectures in China for the time-series analysis. As shown in Figure 10 and Table 3, the goodness of fit *R*^2^ of the double-logarithm regression model for the prefectural scale was greater than 0.90 for the top 250 prefectures from 2012 to 2018, which passed the significance test. The goodness of fit was significantly better than for all prefectures. In the ln-ln model, the regression line hardly changed from 2012 to 2018. In the double-logarithm regression model, the value of *q* was less than 1.0. As the *q* values were all less than 1, this indicates that among the top 250 prefectures, the monopolistic nature of urban development in the first prefecture was not prominent enough, and the scale of urban development in the small- and medium-sized prefectures was not much different and the number was large; additionally, the urban development scale of each one was evenly distributed. In addition, the overall *q* value showed a decreasing trend, indicating that the urban development disparity among the top 250 prefectures in China was gradually decreasing, and the urban development scale was more dispersed than concentrated. The urban system changed from decentralized to more decentralized.

From the above statistical analysis, the development of the top 250 prefectures in China was undoubtedly a relative equilibrium distribution type. At the national prefecture-level city scale, taking Beijing for example, in 2012, Shanghai’s GDP was 1.13 times Beijing’s GDP, Chongqing’s GDP was 0.56 times Beijing’s GDP, Chengdu’s GDP was 0.46 times Beijing’s GDP, and Lhasa’s GDP was 0.01 times Beijing’s GDP. However, with the advancement of urban development, Shanghai’s GDP was 1.09 times Beijing’s GDP, Chongqing’s GDP was 0.69 times Beijing’s GDP, Chengdu was 0.50 times Beijing’s GDP, and Lhasa was 0.02 times Beijing’s GDP. Lhasa’s GDP in 2012 was 1.84 times that in 2017. The gaps between several cities were constantly decreasing. The decreasing of the *q* value means that cities also grew as the built-up areas expanded, the population increased, and the economy grew, which resulted in the narrowing of the gap between the prefectures. The state provided financial and policy support to these underdeveloped regions to improve the construction of service facilities in these regions and develop characteristic industries. In recent years, with the implementation of policies, such as the revitalization of northeast China, the rise of central China, and the development of western China, the provincial capitals and port cities in northeast China and central and western China had become the focus of economic growth. For instance, the development of the cultural tourism industry in Tibet and the contribution of the cultural industry to the economic growth in Tibet were becoming increasingly obvious. The cities in these locations, regardless of their size, had a significant concentration of industries and capital, abundant employment opportunities, and absolute advantages in terms of infrastructure and public service facilities, forming strong incentives for the gathering of a floating population and promoting the development of the urban scale and level. The Pearl River delta, the Yangtze River delta and the west coast of the straits in the southeast were the regions with the highest concentration of floating population in China [1]. Therefore, the prefectures in these locations were more likely to have opportunities for development and mainly clustered in the middle-rank, which implies that China’s current prefectural city development strategy focused on small- and medium-sized prefectures by developing the prefectures at the top of the regional urban development, which radiated to the development of the remaining cities.

#### 4.2.3. Urban Development at the County Level

**Figure 11:**
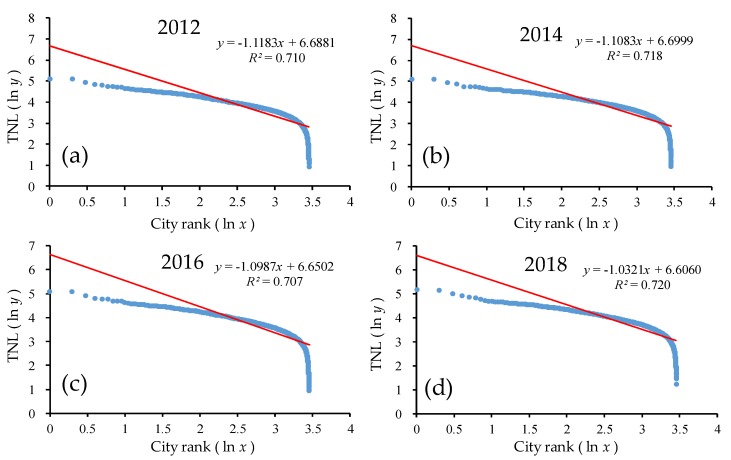
The ln-ln plot of the rank–size of the urban development for all counties. Line regression of (**a**) TNL and ranking of all counties in 2012; (**b**) TNL and ranking of all counties in 2014; (**c**) TNL and ranking of all counties in 2016; (**d**) TNL and ranking of all counties in 2018.

As shown in Figure 11 and Table 4, the goodness of fit *R*^2^ of the double-logarithm regression model for the provincial scale was greater than 0.70 for all provinces from 2012 to 2018, which passed the significance test. In the double-logarithm regression model, the value of *q* was greater than 1. The change of the *q* value in the county-level cities fluctuated slightly from 1.12 in 2012 to 1.03 in 2015, and increased slightly by 0.01 from 2015 to 2018, showing a decreasing trend overall and M-shaped volatility evolution characteristics. On the one hand, this shows that urban development at the county-level in China was a primate distribution (*q* > 1) and it meant that in the urban system, the development of the counties was concentrated mainly in large counties, and the development in medium and small counties was relatively insufficient. Moreover, the greater the *q* was, the stronger the monopoly capacity of the primate county was. On the other hand, the decrease of the *q* value from 2012 to 2015 indicates that the overall trend of the urban development scale on the county-level scale in China was increasingly dispersed, and the power of the urban development scale distribution tended to be dispersed more than it was concentrated. The urban system changed from decentralized to more decentralized. The counties in the lower rank developed rapidly, while those in the higher rank developed slowly. The increase of the *q* value from 2015 to 2018 indicates that the overall trend of the urban development at the county-level scale in China was increasingly balanced, and the power of the urban development scale distribution tended to be concentrated more that it was dispersed. As the urban system changed from decentralized to centralized, the development speed of cities in the back of the sequence slowed down, while the development speed of cities in the front accelerated. In general, from 2012 to 2018, the scale of urban development was more dispersed than concentrated.

As shown from Figure 11, in the ln-ln model scatterplot, there was also an obvious trailing phenomenon, which significantly deviated from the regression line. In addition, the rear part of the scale distribution curve of the urban development level for the county deviated from Zipf’s law. The counties located in western China had small populations and had underdeveloped urban development systems and inconvenient traffic, while the counties in the eastern coastal area were concentrated, and their superior geographical location attracted foreign investment. Additionally, the implementation of national economic policies and the radiating role of several major coastal urban agglomerations caused the eastern counties to be more prosperous than the western counties.

**Figure 12:**
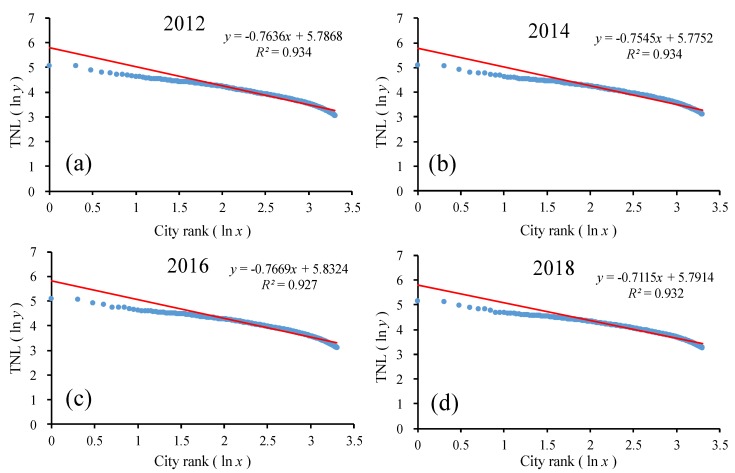
The ln-ln plot of the rank–size of the urban development for the top 2000 counties. Line regression of (**a**) TNL and ranking of the top 2000 counties in 2012; (**b**) TNL and ranking of the top 2000 counties in 2014; (**c**) TNL and ranking of the top 2000 counties in 2016; (**d**) TNL and ranking of the top 2000 counties in 2018.

On the same scale, this study chose the top 2000 counties in China for the time-series analysis. As shown in Figure 12 and Table 4, the goodness of fit *R*^2^ of the double-logarithm regression model for the county-level scale was greater than 0.90 in the top 2000 counties from 2012 to 2018, which passed the significance test. In the ln-ln model, the regression line hardly changed from 2012 to 2018. In the double-logarithm regression model, the value of *q* was less than 1. As the *q* values were all less than 1, this indicates that among the top 2000 counties, the monopolistic nature of nighttime lights in the first county was not prominent enough, and the scale of nighttime lights in small- and medium-sized cities was not much different and the number was large; additionally, the urban development scale of each one was evenly distributed. Additionally, the overall *q* value showed a decreasing trend, indicating that the urban development disparity among the top 2000 counties in China was gradually decreasing, and the urban development scale was more dispersed than concentrated. The urban system changed from decentralized to more decentralized.

From the above statistical analysis, the development of the top 2000 counties in China was undoubtedly a relative equilibrium distribution type. However, when considering all counties, the scale of urban development was concentrated, where large counties were prominent and small and medium counties were not sufficiently developed. The decreasing *q* value means the gap between the counties had narrowed and the small and medium counties tended to be more developed in China between 2012 and 2018. The actual scale of China’s top-ranking counties, especially the largest counties, was smaller than the theoretical value. It can be seen that China’s current county development strategy focused on the top counties. Therefore, while grasping the differentiation characteristics of the national zones, we should pay attention to the urban development model of counties and cities, improve the radiation effect and weaken the polarization effect in the central area zone, strengthen the construction of central towns in low-level development zones in the central and western regions, and realize the development of elements along the road. The agglomeration of the axes, through the development of the towns and cities and the expansion of the radiating force, improved the neighborhood development environment of low-level counties and cities, optimized the urban functions of the eastern coastal areas, enhanced the industrial structure, adjusted the regional spatial structure, and coordinated the central county and surrounding counties. The development of the county would strengthen its radiation-driven ability toward regional economic development.

#### 4.2.4. Comparison of Urban Development at Different Scales

**Figure 13:**
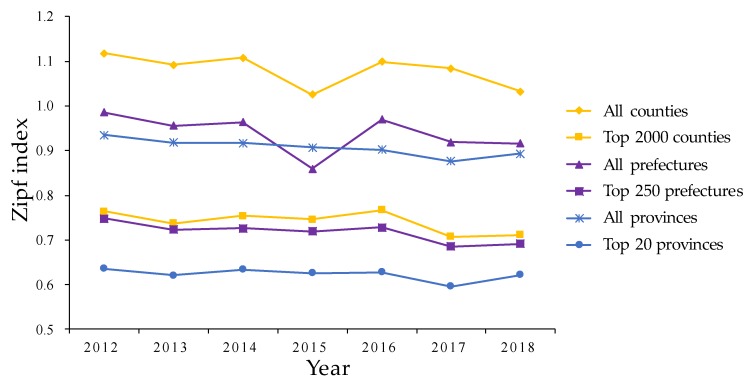
The Zipf index of urban development at multiple scales.

China’s administrative structure has a multiple-system pattern, where a provincial unit is directly composed of several prefectural units and a prefectural unit is composed of several county-level units. A provincial unit generally has stronger administrative, social, demographic, and economic powers than a prefectural unit and county-level unit, probably leading to a differential impact on the *q* value. This study involved a thorough analysis of urban development at provincial, prefectural, and county levels for an improved understanding of how spatial scale change influences urban development. Since single-level studies cannot represent spatial patterns in other levels [58], we needed to adequately and explicitly explore and compare the *q* value at different levels (Figure 13). Our results showed that there were different spatiotemporal variations of urban development within different scales. Since there was not much difference, with a relatively even equilibrium state between the top 20 provinces, 250 prefectures, and 3000 counties, *q* was less than 1. The Zipf index distribution pattern was generally present in all provinces, prefectures, and counties, while the *q* value was inconsistent due to different scales. The *q* value when considering all counties was larger than 1.0 but the *q* values when considering all provinces and prefectures were all less than but near 1.0, while the trend of the *q* valve at three different levels was all decreasing. Meanwhile, the *q* values of all prefectures were higher than in all provinces. The urban development of counties was more concentrated, while the urban development of prefectures and provinces was relatively close to the ideal state of Zipf’s law. This implied that the scale change strongly affected the spatiotemporal variations of urban development in China, i.e., the smaller the administrative scale was, the greater the difference of urban development was [35].

Urban development in China was highly sensitive to changes in the spatial scale. Spatial differentiation resulted in different regional urban development on different scales in China. Spatial differentiation resulted from different levels of administrative divisions in different responses to the guidance and planning policies of national regional urban development. This spatial effect was a combination of top-down national strategies and bottom-up local responses [49]. In their study, Fang et al. [59] verified that China’s city sizes became more evenly distributed before 2000, and this pattern was reversed after 2000, as evidenced by the Zipf’s law index using city-level data from 1949 to 2012. In addition, Wang et al. [60] proved that the urban development of prefectures in the Chengdu–Chongqing urban agglomeration gradually evolved from the unbalanced to the balanced state over time, and meanwhile, urban development gradually shifted from big cities to small- and medium-sized cities. The distribution of urban development tended to shift from big cities to small- and medium-sized cities, which was also evidenced in our study, though our objects were different. The development of various cities was relatively balanced. Because provincial and prefectural scales were larger and fewer, provincial and prefectural scales could not have a very high *q* value compared with county scales. This showed that in terms of urban development in China, the smaller the spatial scale was, the easier it was to show the difference in urban development.

At the same time, policy changes would have an impact on urban development at different scales [61,62]. The national urban development strategy underwent a major transformation from small- and medium-sized cities with key development aimed toward a coordinated urban pattern relying on city clusters. With China’s development entering a new stage, people were increasingly concentrated in metropolises. Therefore, the national urban development strategy also came into being due to the change of time. From the guideline of the 1980s Rein center city scale, it developed the medium-sized city proper, actively developed small cities, and had a focus on the development of small towns. By the year 2014, suggestions were put forward for the new urbanization planning to strengthen the function of central city radiation; speed up the development of small- and medium-sized cities, with a focus on the development of small towns; and promoting the coordinated development of big and medium cities and small towns. In 2011, China’s 12th Five-Year Plan (2011–2015) had proposed to take a diversified urbanization path that suited the coordinated development of large, medium, and small cities, as well as small towns, in line with China’s national conditions, and to become a new driving force for the change of China’s urban structure. In 2016, China’s 13th Five-Year Plan (2016–2020) proposed to promote the development of urban agglomerations and strengthened the radiation effects of core cities and accelerate the development of small and medium cities, as well as some select towns. In the report of the 19th National Congress of the Communist Party of China (CPC), it was recently put forward that urban patterns for the coordinated development of large, medium, and small cities and small towns should be built with urban agglomeration as the main focus. In the study conducted by Wan et al. [63], they found that the growing distance between the distribution of urban development in China and Zipf’s law was consistent with the policy direction of the central government’s relevant five-year plan, with government intervention helping to expand and market forces helping to reduce the numerical deviation of Zipf’s law in cities. Therefore, the *q* value generally decreased, reflecting that the cities’ urban development in the middle and low ranks had increased continuously and showing that national urban development policies had worked. However, the *q* value decreased to different degrees in different scales due to their different influences regarding implementing policies. For the three scales over seven years, the *q* value, along with the social, economic, cultural, ecological environment, and population grew differently at different scale perspectives, and the development of the city presented a decreasing process differently for different scale perspectives, but the trend was consistent. China’s urban development scale distribution was stable, in line with Zipf’s law and was more dispersed; from 2012 to approximately 2017, the overall trend fluctuated toward diversification, followed by a trend toward balance. The distribution fluctuation of the urban development scale had a strong coupling with China’s urban development policy, regional economic policy, and economic development.

### 4.3. Limitations and Future Perspectives

This study explored whether China’s urban development satisfies Zipf’s law with a multiscale perspective from the NPP-VIIRS data. However, there were still some limitations. First, the method of correction of the NPP-VIIRS data needs to be improved and the accuracy of the corrected NPP-VIIRS data could be higher. The accuracy errors and data volatility had a certain impact on the study results. In a future study, we could combine the multi-temporal noctilucent remote sensing image correction method, using calibration view data [64] and combining high-resolution remote sensing images and other multisource data with traditional methods of correction of the NPP-VIIRS data to enhance the accuracy. Second, the radiance of the NPP-VIIRS characterized urban development, though it needs to improve because the NPP-VIIRS data lacks spatiotemporal continuity, which means the NPP-VIIRS data sets for the different consumption habits and different countries has different forms. For example, the increase of nighttime light in politically stable countries generally indicates urban expansion, while the increase of nighttime light in politically turbulent countries generally indicates the return of refugees; the expansion of built-up areas in developed countries generally leads to an increase in the area lit at night, and some developing countries have limited power supply capacity. The expansion of built-up areas can sometimes not lead to an increase in the area lit at night. When using luminous remote sensing for geoscience and socio-economic knowledge discovery, a more cautious attitude should be adopted, and the social, economic, cultural, and even religious background of the study area needs to be considered to a greater extent. Choosing the right spatial scale is also important for noctilucent remote sensing research. Furthermore, we used big data [65] and nighttime light data to study urban development. Considering the fusion of multi-source data, the nightlight images have been combined with land surface temperature [66,67], normalized difference building index [68,69], point of interest [70,71], open street map [72,73], and other relevant data [74,75] to further improve the accuracy of the research data. It is no longer appropriate to use Zipf’s law alone to analyze urban development. Third, this study chose Zipf’s law to analyze a single aspect of China’s urban development. The next step is to combine other methods (such as the Markov chain, fractal dimension, and the urban pyramids) to analyze the characteristics of different scales of urban development (dynamic evolution of the urban development scale) on a more microscopic scale and then explore the factors behind urban development. Finally, the *q* value was not fully diagnosed at multiple scales. In the future, an in-depth analysis of diagnostics tests will be investigated. The root-mean-square error or mean absolute percentage error will be calculated to measure the deviation between the optimal value of the *q* value and the value obtained by the counties, prefectures, and provinces, making the analysis results more reliable.

## 5. Conclusions and Policy Implications

This study has attempted to explore whether China’s urban development satisfied Zipf’s law at multiple scales using the NPP-VIIRS data from 2012 to 2018. After correction of the NPP-VIIRS data, the fitting degree between the total nighttime light value and the statistical data (GDP, EPC, and population) became higher, with an average *R*^2^ value of 0.74. Zipf’s law was then employed to synthetically evaluate urban development. The Zipf distribution pattern was generally presented in all provinces, prefectures, and counties. Differences regarding the Zipf exponents was analyzed from different perspectives. The developments of the top 20 provinces, top 250 prefectures, and top 2000 counties in China were undoubtedly a relatively even equilibrium distribution type, while the developments of all provinces, all prefectures, and all counties experienced tail-dropping from 2012 to 2018. The value of *q* was between 0.99 and 0.92 in all prefectures and between 0.94 and 0.89 in all provinces, which were close to the ideal state according to Zipf’s law. While the value of *q* was from 1.12 to 1.03 in all counties, it showed the primate county with the stronger monopoly capacity converting to the ideal state according to Zipf’s law with an average *R*^2^ value of more than 0.70. The value of *q* was between 0.76 and 0.71 in the top 2000 counties, between 0.75 and 0.70 in the top 250 prefectures, and between 0.64 and 0.62 in the top 20 provinces; therefore, there was little difference in the scale of urban development within the urban system at the multiscale levels, with an average *R*^2^ value greater than 0.90. Additionally, the *q* value appeared to decrease for multiple scales, which showed that the gap between provinces, prefectures, and counties had narrowed. For three multiple-scale cities, the Zipf exponents evolved in a decreasing process within the 7 years as the metropolitan development grew through an increasing process involving societal, economic, cultural, ecological environment, and population development.

Our results have implications for China’s urban development from a multiscale perspective. Different scales led to different results. Hence, the urban development analysis results of Zipf’s law are different at different scales, which are conducive to comprehensive analysis and analyzing the change of urban development in the country from various perspectives. By properly monitoring the dynamic changes of urban development, the government could make reasonable adjustments to the implementation of urban development strategies and policies; lead urban development to a more balanced path; and reduce the gap between provinces, cities, and counties at the same level. However, how should the development policy be adjusted? In theory, according to Zipf’s law, when *q* = 1, the urban development system reaches the optimal scale distribution at multiple scales, with the highest development benefit and the most reasonable distribution of material and energy consumption. In the future, with the rapid development of Chinese cities, the mutual radiation between subregions will be enhanced. Therefore, the policy is improved according to the *q* value. The nighttime light data provide a good means to detect the dynamic changes of urban development in China at different scales. The use of nighttime light data, combined with existing socioeconomic statistics and other types of remote sensing data, can be used to obtain the development situation of the country’s provinces, cities, and counties; monitor national development trends; provide support for regional resource allocation and social assessment; and provide effective assistance for government policy formulation. Considering the social and economic differences among regions, different urban development strategies are needed for different regions to cope with their urban development statuses and make China’s urban development sustainable. Therefore, when we formulate urban development policies at various scales, we should neither unilaterally emphasize the development of megacities and large cities, nor emphasize the development of small cities. Instead, we should carry out rational policy-based regulations based on fully grasping the current characteristics of the urban development scale distribution at various scales and its evolution law.

Existing environmental and public health problems are closely related to the modes of production and lifestyle on which urban development activities depend on. Atmospheric pollutants, such as PM_2.5_ emissions, water pollution, and other environmental and public health problems caused by the human factor when accelerating the urban development processes affect public health. Lu et al. [43] estimated the trend of PM_2.5_ concentrations and its adverse health effects in China and revealed the impacts of urbanization on PM_2.5_ concentration and mortality. Ali et al. [76] showed that urbanization was found to enhance carbon emissions, both in the long run and short run. Therefore, in order to effectively analyze the change of urban development to achieve sustainable urban development and the remediation of environmental problems, the Chinese government should consider multi-dimensional development goals, including economic growth, environmental protection, ecological restoration, and the sustainable use of resources. In the future, urban development should fully consider the spatial distribution between cities, the change of urban spatial characteristics, and the regulation of urban development speed to minimize the damage to the environment caused by urban development and construction and to achieve sustainable development and healthy development.

## Figures and Tables

**Table 1:** Regression results for the corrected and original NPP-VIIRS data. Noted: Mean represented the yearly composite mean data; Max represented the yearly composite maximum data. Avg represented the average results.

Year	The Corrected NPP-VIIRS Data						The Original NPP-VIIRS Data					
	GDP		EPC		Population		GDP		EPC		Population	
	Mean	Max	Mean	Max	Mean	Max	Mean	Max	Mean	Max	Mean	Max
	*R* ^2^	*R* ^2^	*R* ^2^	*R* ^2^	*R* ^2^	*R* ^2^	*R* ^2^	*R* ^2^	*R* ^2^	*R* ^2^	*R* ^2^	*R* ^2^
2012	0.849	0.817	0.891	0.870	0.545	0.574	0.740	0.689	0.785	0.750	0.464	0.437
2013	0.854	0.786	0.883	0.841	0.569	0.565	0.749	0.661	0.781	0.728	0.446	0.444
2014	0.842	0.764	0.901	0.855	0.551	0.546	0.735	0.635	0.798	0.739	0.452	0.423
2015	0.842	0.750	0.874	0.815	0.555	0.555	0.738	0.634	0.773	0.708	0.464	0.443
2016	0.831	0.764	0.896	0.873	0.568	0.557	0.728	0.650	0.795	0.767	0.464	0.438
2017	0.837	0.787	0.905	0.873	0.607	0.612	0.736	0.679	0.805	0.770	0.511	0.505
Avg	0.843	0.778	0.892	0.855	0.566	0.568	0.738	0.658	0.790	0.744	0.467	0.448

**Table 2:** Zipf’s law analysis at the provincial level in China from 2012 to 2018.

Year	All Provinces			Top 20 Provinces		
	$ln\left(S_{i}\right)=ln\left(S_{1}\right)-qln\left(R_{i}\right)$	*R* ^2^	*q*	$ln\left(S_{i}\right)=ln\left(S_{1}\right)-qln\left(R_{i}\right)$	*R* ^2^	*q*
2012	*y* = −0.9348*x* + 6.4720	0.733	0.9348	*y* =−0.6360*x* + 6.2679	0.941	0.6360
2013	*y* = −0.9182*x* + 6.4863	0.728	0.9182	*y* = −0.6212*x* + 6.2833	0.955	0.6212
2014	*y* = −0.9173*x* + 6.4701	0.734	0.9173	*y* = −0.6339*x* + 6.2761	0.957	0.6339
2015	*y* = −0.9071*x* + 6.4715	0.746	0.9071	*y* = −0.6256*x* + 6.2792	0.969	0.6256
2016	*y* = −0.9018*x* + 6.4830	0.736	0.9018	*y* = −0.6275*x* + 6.2947	0.967	0.6275
2017	*y* = −0.8759*x* +6.5357	0.718	0.8759	*y* = −0.5955*x* + 6.3433	0.964	0.5955
2018	*y* = −0.8934*x* + 6.5762	0.739	0.8934	*y* = −0.6218*x* + 6.3901	0.966	0.6218

**Table 3:** The Zipf law analysis at the prefectural level in China from 2012 to 2018.

Year	All Prefectures			Top 250 Prefectures		
	$ln\left(S_{i}\right)=ln\left(S_{1}\right)-qln\left(R_{i}\right)$	*R* ^2^	*q*	$ln\left(S_{i}\right)=ln\left(S_{1}\right)-qln\left(R_{i}\right)$	*R* ^2^	*q*
2012	*y* = −0.9857*x* + 6.3815	0.748	0.9857	*y* = −0.7481*x* + 5.9886	0.945	0.7481
2013	*y* = −0.9555*x* + 6.3570	0.735	0.9555	*y* = −0.7233*x* + 5.9725	0.955	0.7233
2014	*y* = −0.9638*x* + 6.3560	0.737	0.9638	*y* = −0.7265*x* + 5.9632	0.952	0.7265
2015	*y* = −0.8597*x* + 6.1438	0.644	0.8597	*y* = −0.7190*x* + 5.9593	0.953	0.7190
2016	*y* = −0.9696*x* + 6.3898	0.728	0.9696	*y* = −0.7279*x* + 5.9897	0.954	0.7279
2017	*y* = −0.9186*x* + 6.3787	0.737	0.9186	*y* = −0.6859*x* + 5.9942	0.949	0.6859
2018	*y* = −0.9164*x* + 6.4011	0.743	0.9164	*y* = −0.6912*x* + 6.0286	0.945	0.6912

**Table 4:** The Zipf’s law analysis at the county level in China from 2012 to 2018.

Year	All Counties			Top 2000 Counties		
	$ln\left(S_{i}\right)=ln\left(S_{1}\right)-qln\left(R_{i}\right)\;$	*R* ^2^	*q*	$ln\left(S_{i}\right)=ln\left(S_{1}\right)-qln\left(R_{i}\right)\;$	*R* ^2^	*q*
2012	*y* = −1.1183*x* + 6.6881	0.710	1.1183	*y* = −0.7636*x* + 5.7868	0.934	0.7636
2013	*y* = −1.0776*x* + 6.6113	0.705	1.0917	y = −0.7375*x* + 5.7466	0.939	0.7375
2014	*y* = −1.1083*x* + 6.7362	0.718	1.1083	*y* = −0.7545*x* + 5.7752	0.934	0.7545
2015	*y* = −1.0259*x* + 6.6863	0.717	1.0259	y = −0.7459*x* + 5.7625	0.935	0.7459
2016	*y* = −1.0987*x* + 6.6863	0.707	1.0987	y = −0.7669*x* + 5.8324	0.927	0.7669
2017	*y* = −1.0840*x* + 6.6217	0.718	1.0840	y = −0.7070*x* + 5.7539	0.933	0.7070
2018	*y* = −1.0321*x* + 6.6060	0.720	1.0321	*y* = −0.7115*x* + 5.7914	0.932	0.7115
